# The application of long-read sequencing in clinical settings

**DOI:** 10.1186/s40246-023-00522-3

**Published:** 2023-08-08

**Authors:** Josephine B. Oehler, Helen Wright, Zornitza Stark, Andrew J. Mallett, Ulf Schmitz

**Affiliations:** 1 Biomedical Sciences and Molecular Biology, College of Public Health, Medical & Vet Sciences, James Cook University, Townsville, Australia; 2 College of Medicine and Dentistry, James Cook University, Townsville, Australia; 3 Nursing and Midwifery, College of Healthcare Sciences, James Cook University, Townsville, Australia; 4 Victorian Clinical Genetics Services, Murdoch Children’s Research Institute, Melbourne, Australia; 5 University of Melbourne, Melbourne, Australia; 6 Australian Genomics, Melbourne, Australia; 7 Department of Renal Medicine, Townsville University Hospital, Townsville, Australia; 8 Institute for Molecular Bioscience, The University of Queensland, Brisbane, Australia; 9 Faculty of Medicine, The University of Queensland, Brisbane, Australia; 10 Centre for Tropical Bioinformatics and Molecular Biology, Australian Institute of Tropical Health and Medicine, James Cook University, Cairns, Australia; 11 Computational BioMedicine Lab Centenary Institute, The University of Sydney, Camperdown, Australia; 12 Faculty of Medicine & Health, The University of Sydney, Camperdown, Australia

## Abstract

Long-read DNA sequencing technologies have been rapidly evolving in recent years, and their ability to assess large and complex regions of the genome makes them ideal for clinical applications in molecular diagnosis and therapy selection, thereby providing a valuable tool for precision medicine. In the third-generation sequencing duopoly, Oxford Nanopore Technologies and Pacific Biosciences work towards increasing the accuracy, throughput, and portability of long-read sequencing methods while trying to keep costs low. These trades have made long-read sequencing an attractive tool for use in research and clinical settings. This article provides an overview of current clinical applications and limitations of long-read sequencing and explores its potential for point-of-care testing and health care in remote settings.

## Background

First- and second-generation short-read sequencing (SRS) has been the gold standard for genetic profiling in the last two decades [1, 2]. Short-read sequencing, providing reads of typically 150 bp in length, is supported by a wide range of platforms, dominated by Illumina’s fleet of instruments (NovaSeq, HiSeq, NextSeq, and MiSeq), and is valued for its high accuracy and relative cost-effectiveness. However, sequencing small DNA fragments requires complex algorithms for the reconstruction of longer sequence contigs, potentially resulting in inaccurate and incomplete genome assemblies. This can be further compounded in capture-based sequencing such as exome sequencing where there are inherent limitations in identifying and characterising structural variants. These have limited the usage of short-read sequencing for the analysis of complex genomic loci, repetitive elements, or variant phasing (haplotyping) in genetic conditions such as short tandem repeat (STR) expansion disorders (e.g. Huntington’s disease and Fragile X syndrome) [3]. Furthermore, the PCR amplification of sequencing templates can generate artefacts and prohibits the detection of native base modifications [4].

These limitations were the motivation and driving force for the development and application of long-read sequencing (LRS), also known as third-generation sequencing, with the ability to generate sequence reads tens of thousands of bases in length. The LRS process occurs in real time, and both the sequencing and library preparation are conducted without the need for PCR amplification, therefore being free from any PCR-related bias. Also, with the DNA remaining in its native state, LRS technologies can detect base modifications such as methylation directly, without the base conversions required for SRS which are known to degrade DNA [5].

**Fig. 1:**
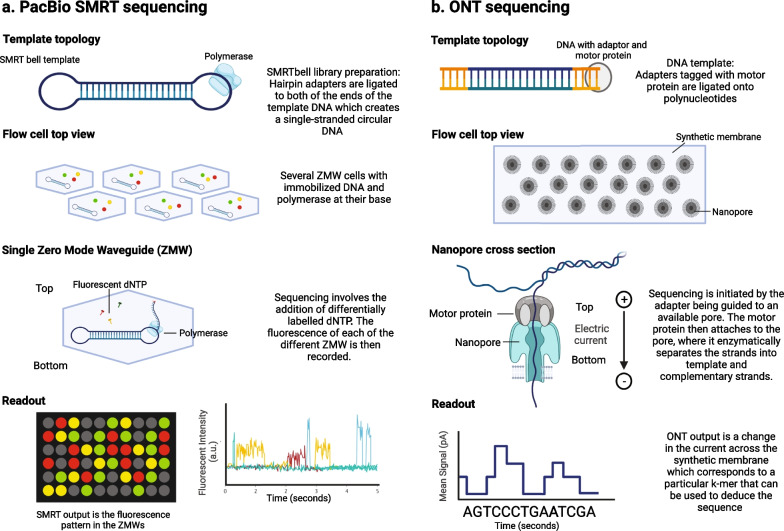
Schematic diagram of **a** PacBio SMRT sequencing and **b** ONT. Created with BioRender.com

Currently, the two main technologies for LRS are Pacific Biosciences’ (PacBio) single-molecule real-time (SMRT) sequencing and Oxford Nanopore Technologies’ (ONT) nanopore sequencing [6] (see Fig. 1).

PacBio’s SMRT sequencing technology was the first LRS technology to reach widespread utilisation [7]. The SMRT technology implemented by their Sequel and Revio platforms utilises parallel systems of polymerase which is bound to DNA circularised to include hairpin sequencing adaptors [8]. The incorporation of labelled bases by the polymerase enables the DNA to fluoresce. The resulting fluorescence is detected using a zero-mode waveguide and a charge-coupled device camera. One limitation of this technology is its error rate of 1–5% in comparison with short-read sequencing which has an error rate of 0.24 ± 0.06% per base [4]. However, this limitation has been addressed by PacBio with HiFi reads, which are based on the circular consensus sequencing concept (reducing the error rate to between 0.1 and 0.5%, i.e. > Q30), thus making the error rate comparable to Illumina sequencing [2].

The latest PacBio sequencing platform, the Revio system, now delivers up to 360 Gb of HiFi reads per day, equivalent to ~ 1300 human whole genomes per year [9]. With improved sequencing chemistry and the help of deep learning algorithms, the Revio system provides significantly improved read accuracy (Q33) and methylation calling capability.

The next successful LRS technology was established by Oxford Nanopore Technologies (ONT). The idea of nanopore sequencing dates back to the 1990s, when Branton and Deamer [10] developed the concept that if bases induce different ionic current bursts during DNA traversing through a tiny channel, it would constitute a new sequencing technique. In 1993, Deamer and colleagues employed α-haemolysin (α-HL), a toxic pore-forming protein secreted by *Staphylococcus aureus*, to manipulate a lipid bilayer and detected DNA translocation through an α-HL nanopore [11]. This became the first nanopore shown to detect recognisable ionic current blockades by both RNA and DNA homopolymers.

Nanopore sequencers (MinION, GridION, and PromethION) and their application in basic and clinical research have undergone substantial growth since ONT provided the first commercially available nanopore sequencer in 2014 [12]. Their technology utilises a membrane that has a grid of embedded nanopores [13]. The membrane separates two ionic solutions which allows an electrical current to flow through the nanopores. During the library preparation process, motor protein-tagged adapters are attached to polynucleotides. These adapters are then guided to available pores to initiate sequencing. Once attached, the motor protein separates the polynucleotide strands into template and complementary strands enzymatically. The template strand is then pulled through the pore by the potential difference across the membrane, often releasing the complementary strand. However, sometimes both strands are pulled through, resulting in a duplex read. As the polynucleotide passes through the pore, there is a unique disruption in current flow for each *k-*mer of consecutive nucleotides [14].

ONT has also made significant innovations in long-read sequencing. One notable advancement is the introduction of adaptive sampling, a computational enrichment technique [15]. Adaptive sampling leverages real-time analysis of the sequencing data to dynamically adjust the sequencing parameters, focusing sequencing efforts on regions of interest. This technique improves the efficiency of data generation by targeting specific genomic regions, allowing for deeper coverage and more effective utilisation of sequencing resources. Adaptive sampling has the potential to enhance the speed, accuracy, and cost-effectiveness of long-read sequencing, opening new possibilities for its implementation in various genomic applications.

Nanopore sequencing also allows for the identification of base modifications both at DNA and RNA level. Modified bases such as 5-methylcytosine in DNA or N6-methyladenine in RNA sequences can be detected based on differences in the current flow (i.e. the squiggle signal) between modified and unmodified bases [16]. The higher density of modified bases makes long-read sequencing particularly valuable for haplotype-resolved mutation and methylation calling, leading to a more comprehensive understanding of the genetic variations and their association with phenotypic traits.

ONT continuously develops new, improved chemistries for their sequencing kits with the latest being the V14 chemistry [17]. The V14 kit chemistry with the R10.14.1 pore leverages enhanced base-calling algorithms and improved signal processing techniques, resulting in reduced error rates and an improved sequencing accuracy (Q20 +) [18]. By minimising sequencing errors, the V14 chemistry enhances the ability to confidently detect genetic variations, structural rearrangements, and other genomic features.

Overall, due to its unparalleled utility in reading genomes, transcriptomes and epigenomes, providing major opportunities for all areas of medical and biological research, LRS was named “Method of the Year 2022” by the journal Nature Methods [19]. This article provides an overview of the current and future applications of LRS in clinical and remote settings, including the use of LRS for diagnosis, prognosis, and therapy selection for constitutional disorders, oncology, transplantation, and infectious diseases. It also aims to critically assess the current challenges of implementing LRS in patient care.

## Clinical applications

### Rare disease diagnosis

Genomic testing in clinical settings is used to identify underlying genetic causes and inheritance of diseases. However, about 50% of all suspected Mendelian conditions remain undiagnosed [20] thereby limiting physicians’ ability to pinpoint the exact underlying mechanism of the disease, provide accurate information to families, or utilise state-of-the-art treatments and improve patient outcomes, and result in extended “diagnostic odysseys” [21]. The current diagnostic shortcomings of genomic testing are in part caused by technical limitations of short-read sequencing in identifying complex structural variants (SVs), sequencing repetitive regions, phasing of alleles, and distinguishing highly homologous genomic regions.

Recent research has shown that LRS technology may play an important role in discovering novel pathogenic mutations in human diseases with a previously unknown underlying genetic cause. A pilot study conducted by Stevanovski et al. [22] demonstrated the utility of nanopore sequencing for the genetic diagnosis of short tandem repeat (STR) expansion disorders [22]. STR disorders are highly prevalent in neurological diseases. Unlike previous studies, their approach enabled unbiased sizing and sequence determination of all known neuropathogenic STR sites in a single targeted assay. Thereby, they were able to identify causes for disorders such as Huntington’s disease, Fragile X syndrome, cerebellar ataxia, and vestibular areflexia syndrome using the same assay [22]. Knowledge about individual STR expansions may reveal previously unknown genotype–phenotype correlations, enabling a better understanding of pathomechanisms and facilitating evidence-based treatment design. Detection of complex and previously unknown genomic variants via LRS could mark the end the long period of time it can take for a patient to receive a diagnosis for their condition in rare neurological disorders, often including multiple clinical evaluations, laboratory tests, and short-read sequencing [22]. It can facilitate earlier predictive testing in at-risk family members, prognostication, informed reproductive decision-making, and enable timely delivery of targeted treatments with improved clinical outcomes.

To further identify and diagnose rare genetic diseases within the population, there has been a drive for the implementation of whole-genome sequencing (WGS) for screening and diagnosis of genetic conditions [23]. The first LRS of the whole genome in a clinical setting was reported in 2017 by researchers at Stanford Medical School who used it to identify the genetic basis of Carney complex, a rare genetic disorder associated with multiple endocrine neoplasia syndrome. Researchers uncovered that the causative mutation was a 2.2-kb deletion in the *PRKAR1* gene [24]. Single-molecule real-time (SMRT) sequencing has also been used to resolve SVs associated with Mendelian disorders [21].

Another promising emerging application of LRS is the diagnosis of genetic conditions such as those affecting colour vision as demonstrated in the study by Haer-Wigman et al. [25]. The genes that are most commonly associated with mild to severe conditions affecting colour vision form the *OPN1LW/OPN1MW* gene cluster. Many individuals with genetic variants in these genes often remain genetically undiagnosed due to the inability of short-read sequencing to differentiate between the highly homologous *OPN1LW* and *OPN1MW* genes. Through the use of copy number analysis via multiplex ligation-dependent probe amplification and sequence analysis via long-read circular consensus sequencing, Haer-Wigman et al*.* were able to identify both structural and nucleotide variants in the *OPN1LW/OPN1MW* gene cluster. Their assay has the potential to be streamlined into clinical settings and improve diagnostic care of individuals with visual impairment.

A recent example of a successful application of LRS to reveal the underlying cause of a rare genetic condition was presented by Viering et al. in Gitelman syndrome. In this rare autosomal-recessive syndrome, biallelic disease-causing *SLC12A3* variants lead to dysfunction of the Na1-Cl2 cotransporter (NCC), resulting in a salt-losing renal tubulopathy characterised by hypokalaemic alkalosis and hypomagnesemia [26, 27]. In approximately 8%–31% of patients with symptoms of Gitelman syndrome only one pathogenic variant of *SLC12A3* is currently found, depending on the techniques used. However, Viering et al. through LRS identified a second likely pathogenic intronic variant in 67% of patients previously identified to apparently harbour only one variant. Those with two *SLC12A3* variants had a more severe electrolyte phenotype than other patients [26]. These findings demonstrate the importance of genetic screening of introns for rare kidney diseases and display how LRS has the capacity to increase diagnostic yield of genomic testing.

Another great potential of long-read sequencing technology lies in the rapid diagnosis of rare genetic diseases to guide the clinical management of critically ill patients. Researchers from Stanford University recently presented an ultra-rapid nanopore whole-genome sequencing workflow that was able to diagnose rare genetic diseases in an average of eight hours. Their pilot study involved 12 critical care patients, five of whom received a genetic diagnosis from the sequencing information, guiding in clinical decision-making such as heart transplantation and change of medication [26]. In one of the cases, it took a swift 5 h and 2 min to sequence the patient’s genome, which set the Guinness World Records title for fastest DNA sequencing technique [28].

### Oncology

Cancer genomics is one of the representative fields in which LRS technologies have already achieved significant results and proven to have considerable clinical potential. SVs in cancer genomes, similar to somatic mutations, can affect the function of oncogenes and tumour suppressor genes. A large number of cancer patients are routinely screened for pathogenic SVs in clinical practice, most often by copy number profiling or karyotyping [29]. Although these methods are robust and relatively cost-efficient, LRS is better at detecting variants such as copy-balanced SVs [30]. They also do not provide base-pair resolution accuracy, or the possibility to resolve complex SVs. By using long reads, it is possible to detect many novel SVs that would otherwise be missed when using a short-read sequencing approach, for example in genes in which other known variants have been shown to substantially increase cancer risk [30]. Although the full functional impact of many of these variants is currently unknown, the detection of additional variants in cancer-relevant genes indicates that current analysis pipelines may often underestimate the mutational burden in tumour samples.

A pioneering study by Norris et al. used MinION LRS to analyse SVs in the *CDKN2A* and *SMAD4* tumour suppressor genes in pancreatic cancer cell lines [31]. From reads of > 500 bp in length, they were able to detect loss-of-function variants in these tumour suppressor genes represented by translocations, inversions, deletions, and the combination of inversions. Nattestad et al. demonstrated the detection of SVs in genomes of a breast cancer cell line utilising both long-read and short-read sequencing [32]. Interestingly, they found that *ERBB2* amplification appeared within complex rearrangements at chromosome 8, which could only be precisely identified by LRS.

Euskirchen et al. developed a method to diagnose central nervous system (CNS) tumours using MinION technology [33]. They implemented a 1-day workflow for the diagnosis of the CNS tumours, which successfully identified diagnostically relevant alterations, such as 1p/19q codeletion and focal amplifications. They also detected the amplification of cancer-related genes, such as *EGFR*, *PDGFRA*, and *CDK4* [33]. This study demonstrated the capacity of LRS to efficiently and effectively identify SVs for the diagnosis of cancer in a clinical setting.

The benefits of a fast turnaround using the portable MinION sequencer were emphasised in several studies testing the diagnostic yield of nanopore sequencing in patients with haematological malignancies, such as chronic lymphocytic leukaemia and chronic myeloid leukaemia [34–36]. In these patients, the MinION was used to detect SVs in the *TP53* and *ABL1* genes. Cumbo et al. developed a nanopore-based assay for rapid (24 h) targeted sequencing of six genes (*NPM1*, *FLT3*, *CEBPA*, *TP53*, *IDH1*, and *IDH2*) that are frequently mutated in acute myeloid leukaemia [37]. The nanopore sequencing-based approach provided significant advantages compared to traditional means of genetic profiling, such as reducing the turnaround time from about 7 days to 24 h and bringing down the costs to approximately USD 200 [37].

Another clinical application of LRS in the field of oncology that has shown significant promise is the multilayer analysis of the transcriptome and the epigenome. Transcriptomics has benefited from the application of LRS as it allows full coverage of transcript sequences, and thus, structures of transcript isoforms can be determined by sequencing full-length complementary DNAs (cDNAs). In the field of oncology, fusion transcripts are of particular interest since they have been identified as major drivers of carcinogenesis [38]. Identification of specific gene fusions can be essential for distinguishing benign from malignant conditions and for the subclassification of neoplasms that may have different management and prognosis.

In a study by Jeck et al., real-time long-read data generated using the MinION sequencing system enabled the identification of *BCR-ABL1* fusion transcripts with > 100 reads within 15 min of sequencing [39]. Their nanopore-based sequencing assay showed that LRS used for fusion transcriptomics may be a valid approach for laboratories with low specimen volume and for clinical cases in need of rapid results. Rapid identification of gene fusions is particularly critical for patients with acute leukaemia who require immediate treatment and where the type of therapy can vary dramatically depending on the leukaemia subtype.

To take full advantage of new LRS technologies for fusion identification and characterisation, fusion detection algorithms have been developed. For example, Davidson et al. developed JAFFAL, a novel pipeline to identify fusions in long-read transcriptome sequencing data [40]. JAFFAL compares cancer transcriptomes to a reference transcriptome and overcomes potentially high error rates of LRS by using alignment methods and filtering heuristics that are designed to manage noisy long reads. This allowed Davidson et al.to identify previously known fusions in primary cell lines of cancer patients and uncover a novel fusion composed of three genes, *BMPR2-TYW5-ALS2CR11*, in the lung cancer cell line H838 [40].

Furthermore, LRS can be applied to detect and screen for epigenetic changes in cancer. Epigenetic changes have important roles in the control of cellular function through transcriptional regulation. Epigenetic alterations can lead to aberrant gene expressions or splicing dysregulation and promote tumorigenesis [41, 42]. The cancer cell genome undergoes dramatic shifts in DNA methylation, including genome-wide hypomethylation in conjunction with local areas of hypermethylation. Hypomethylation can cause the expression of certain genes, such as oncogenes, whereas hypermethylation may inhibit tumour suppressor genes. Currently, the standard method of determining the extent of methylation within the genome is whole-genome bisulphite sequencing [43]. This method does, however, have certain limitations such as it cannot distinguish between the different types of methylation (5mC and 5hmC), and is susceptible to PCR bias and DNA degradation. Nanopore sequencing can overcome these limitations as it can detect the methylation of cytosine directly using signals from a sequencing electrogram without any pre-treatments. While nanopore sequencing has been more widely used, both the ONT and PacBio LRS technologies can be employed for methylation calling [44–46].

Kuschel et al. demonstrated that LRS can be used to determine the methylation of brain tumours and their classification [47]. In their nanopore study, whole-genome sequencing was used for rapid and cost-effective generation of genome-wide 5-methylcytosine profiles as input to supervised classification, using random forests complemented by a medium-resolution copy number profile derived from the same raw data. They managed to discriminate 82 distinct tumour entities from public reference data with a high confidence score. Their approach requires further prospective evaluation but has the potential for clinical implementation.

### Infectious diseases and microbiota

An emerging important clinical application of LRS is its use in infectious diseases. The speed of LRS via ONT platforms, providing results in just a few hours, makes it an attractive option for the in situ diagnosis of infectious pathogens facilitating a potential rapid response for the identification and management of disease sources and disease spread. Real-time genomic surveillance of pathogens has therefore become a new tool to assist difficult epidemiological investigations and to provide an environmental context to emerging infectious diseases. This has the potential to improve the efficiency of resource allocation and the timeliness of epidemiological investigations through genomically informed investigations of transmission chains.

Arguably, the most inspirational clinical application of the MinION sequencer for infectious disease monitoring has been in the Ebola virus outbreak in West Africa in 2015. The portability and capacity to sequence when simply plugged into a laptop allowed real-time genomic surveillance of the epidemic in the field [48, 49]. This was crucial for clarifying patterns of virus evolution, monitoring the validity of diagnostic assays, and investigating transmission chains.

Leading on from the experience in the Ebola outbreak, LRS has also been increasingly employed during the COVID-19 pandemic. As SAR-CoV-2 is a highly infectious virus that is prone to mutations, viral WGS is recognised as a critical tool for studying its transmission and evolution [50]. Nanopore sequencing has frequently been used complementarily and sometimes replaced short-read sequencing as ONT devices can be inexpensive, highly portable, require short library preparation times, enable rapid generation of results with potential real-time data analysis, and require only limited laboratory infrastructure [51]. This allows a faster response to emergent strains of SARS-CoV-2, reducing the risk of a public health crisis. Additionally, nanopore sequencing allows varied levels of throughput, single (e.g. Flongle), multiple (e.g. MinION), or tens/hundreds (e.g. PromethION) of specimens per flow cell [52]. It can therefore strengthen COVID-19 surveillance initiatives by enabling point-of-care WGS analysis and fast turnaround for critical cases, in particular in isolated and under resourced settings, as shown by Smarakoon et al. who developed and employed a nanopore sequencing analysis toolkit for portable Android devices [53]. Nevertheless, while LRS has major benefits, SRS of the whole virus genome with Illumina platforms remains the gold standard for COVID-19 surveillance as several studies have shown that nanopore sequencing can fail in accurately detecting short indels and variants at low read counts [50, 54]. Healthcare facilities can, however, use nanopore sequencing platforms to rapidly diagnose large numbers of patients with limited resources in remote settings and the technology can therefore have an important complementary function in COVID-19 response strategies [55].

More recently, nanopore sequencing has also shown to be a valuable tool in the monitoring and the management of the 2022 monkeypox virus (MPXV) outbreak [56]. As with the COVID-19 pandemic, monkeypox highlighted the global inequities in access to diagnostics and treatments. The complexity of the MPXV genome and requirement of technical laboratory skills and infrastructure (e.g. availability of biosafety level 3 laboratories for virus propagation) and access to sequencing platforms with high sequencing output, such as Illumina, impeded sequencing for many laboratories, especially for those with geographic or economic disadvantage [56]. However, Brinkmann et al. presented an amplicon-based assay for MinION nanopore sequencing, which could overcome these challenges by having the capacity to be scaled up for sequencing in laboratories already performing shotgun sequencing and enables sequencing for laboratories without access to Illumina platforms [57]. Up to 99% of the MPXV genomes could be generated with sequencing of only 200,000 reads. This was also achieved for samples with higher cycle threshold (Ct) values of up to 30, reflecting lower virus load, that are challenging to sequence on Illumina platforms and require an extensive number of reads [57]. This work shows that amplicon-based nanopore sequencing can contribute to solving unanswered questions of the 2022 outbreak and monitor the frequency and appearance of future MPXV mutations while providing a diagnostic assay for underdeveloped countries.

The ability to identify target DNA of pathogens in clinical samples in a timely fashion and not being limited by the length of DNA fragments to be sequenced, has made the MinION sequencer also an attractive technology for expediting malaria elimination and reducing the global malaria burden [58]. Its usage cuts across targeted sequencing, as seen in ultra-deep sequencing of *Kelch13* genes in Plasmodium parasites diagnosing the C580Y mutation of *P. falciparum* and in combination with loop-mediated isothermal amplification in diagnosing human malaria parasites. To achieve malaria elimination, early examination of parasites’ genotypes to avoid the spread of drug-resistant parasite strains and unravelling the disease dynamics at the vector level are important. However, these can only be made possible by a sequencing approach that is accessible, affordable, and easy to use, which is embedded in ONT technology. The technology could therefore be part of the solution to malaria elimination in malaria-endemic regions across low-income countries.

LRS can also make a significant contribution to the treatment and diagnosis of tuberculosis. This was demonstrated in the 2022 study by Gómez-González and colleagues^28^. They utilised the MinION sequencer to identify drug resistance in *M. tuberculosis* to improve its treatment in the field. The MinION sequencer was shown to be a cost-effective way to detect well-established drug resistance variants and undertake phylogenetic reconstruction with potential application in transmission analysis. Their study demonstrated that the MinION portable sequencer represents a feasible cost-effective approach for personalising treatment of infectious diseases in remote settings.

Additionally, hybrid long-read and short-read sequencing is used to leverage benefits and overcome limitations of both technologies in the field of antibiotic resistance research, enabling comprehensive and accurate characterisation of microbial genomes. Several studies have utilised hybrid sequencing to investigate antibiotic resistance mechanisms and identify novel resistance genes. For instance, a study by Marco et al. employed hybrid sequencing to reveal a large diversity of antibiotic resistance in *Mycobacterium tuberculosis* [59]. Similarly, a research article by Khezri et al. utilised hybrid sequencing to identify plasmid-mediated resistance genes in multidrug-resistant *Klebsiella pneumoniae* and *Escherichia coli* isolates [60]. This study revealed that hybrid sequencing provides a better resolution of plasmids from clinical samples and could indirectly shorten the time required to detect pathogenicity factors in clinical settings. Therefore, hybrid assembly has the potential to reveal factors related to microbial pathogenicity in clinical and mixed samples.

Sexually transmitted infections (STIs) remain a major public health problem and are therefore high on the global health agenda [61]. STIs including those caused by *Chlamydia trachomatis, Neisseria gonorrhoeae, Treponema pallidum* and *Trichomonas vaginalis* bacterium or parasites are all curable with existing, effective single-dose antibiotic regimes. However, recently there has been a surge in the number of antimicrobial resistant (AMR) strains of STIs, which has made them difficult to treat and stop their spread [62]. Limitations to current national surveillance systems for reporting AMR trends, alongside a reduction in culture-based diagnostics and susceptibility testing, have further led to an increasing need for rapid diagnosis and identification [63]. An important clinical study by Street et al. utilised the MinION to sequence *N. gonorrhoeae* DNA directly from urine samples from men with symptomatic urethral gonorrhoea to achieve the nearly complete reconstruction of the *N. gonorrhoeae* genome [64]. But the study also revealed that metagenomic sequencing has limitations, such as the potential for contamination, particularly as the approach relies on nonselective amplification of all DNA present.

Another promising application of LRS in the clinical setting is the analysis of microbiota. For example, microbiota of the female reproductive organs can have vast associations with conditions such as genital infections, endometritis, and threatened miscarriage [63]. Komiya and colleagues recently presented a simple workflow for high-resolution and rapid differentiation of vaginal microbiota using the MinION [63]. The process implements a minimally invasive sampling procedure in combination with the MinION applicable to vaginal specimens with a low bacterial load. The analysis also has a very short turnaround time of just 4 h and is economically viable [63]. While this study focused on vaginal microbiota, a similar workflow could be applied to many clinical areas in the future, and the benefits of MinION could make it easier for clinicians to successfully perform bacterial metagenome analyses.

### Transplantation

Long-read sequencing has emerged as a valuable tool in the field of transplantation, particularly in understanding the complex interplay between donor and recipient immune systems. With the advent of PacBio and ONT LRS, researchers have been able to perform highly accurate and comprehensive human leukocyte antigen (HLA) typing. Cornaby et al. utilised LRS to analyse the HLA diversity in a large cohort of transplant patients, shedding light on the importance of donor-recipient matching and its impact on post-transplant outcomes [65].

LRS has revolutionised HLA typing by allowing for the identification of rare and complex HLA alleles, which were previously very hard to detect using traditional SRS methods. For instance, a study by Lehmann et al. employed PacBio LRS to characterise HLA alleles in a cohort of kidney transplant recipients [66]. The researchers found that the presence of certain HLA alleles was associated with an increased risk of acute rejection, providing valuable insights for personalised transplantation strategies. LRS technologies have also facilitated the identification of novel HLA alleles, thereby enhancing our understanding of HLA diversity and its implications in transplantation immunology.

In addition, LRS has played a crucial role in unravelling the causes of graft versus host disease (GVHD). GVHD is a serious complication that can occur after transplantation, where the donor's immune cells attack the recipient's tissues. A recent study by Zhang et al. utilised nanopore sequencing to perform transcriptome analysis of immune cells involved in GVHD [67]. They identified gene expression patterns associated with the development and severity of GVHD, providing valuable insights into the underlying molecular mechanisms. This knowledge has the potential to inform the development of targeted therapies to mitigate GVHD and improve patient outcomes.

Furthermore, LRS has proven instrumental in elucidating the role of killer-cell immunoglobulin-like receptor (KIR) genes in transplantation biology. KIR genes encode receptors expressed on natural killer (NK) cells, which play a crucial role in immune surveillance and regulation. Through LRS researchers have gained a comprehensive understanding of KIR gene diversity and its impact on transplantation outcomes. A notable study by Roe et al. used the technology to investigate KIR gene expression patterns in a cohort of hematopoietic stem cell transplant recipients [68]. The findings revealed associations between specific KIR genotypes and the occurrence of graft rejection.

## Meeting health needs

A major disadvantage of traditional short-read genome sequencing technology has been the need for high capital investment, which resulted in sequencing infrastructure being located in dedicated sequencing centres. Consequently, the shipment of samples can become the most time-consuming step of an investigation, rather than the sequencing or analysis work itself [69]. Research shows that such limitations can be overcome by using portable sequencing technology, such as the MinION. Compared with expensive stationary or bench-top instruments, it is possible to scale this platform out to potentially unlimited numbers of laboratories and locations. Therefore, portable sequencing may soon be used for clinical applications in logistically challenging locations and may thus help address health inequalities. Patient demographics which could benefit most from portable third-generation sequencing are rural and remote populations. Research has consistently shown that residents of rural and remote communities have shorter life expectancies and higher disease-specific mortality, particularly with respect to some cancers, compared to urban residents [70].

Importantly, the utility of portable sequencing has already been proven in field situations, such as diagnostic tent laboratories during the Ebola epidemic. Using the MinION system, it was possible to generate results in under 24 h, with sequencing taking 13–60 min [49]. This showed the tremendous potential and applicability of LRS for genomic surveillance in resource-limited and remote communities.

Additionally, in the field of infectious diseases, whole-genome sequencing has rapidly become the new gold standard in scientific research and a clinical diagnostic tool, particularly in industrialised countries. LRS enhances the detection, diagnosis, and management of infectious parasitic pathogen outbreaks, leading to the containment of many infectious diseases and, thus, improving public health. Such diseases include pneumonia, Ebola, cholera, COVID-19, tuberculosis, and malaria [48, 52, 58, 71]. However, the required infrastructure, including uninterrupted power supply, powerful computers, and modern laboratories, poses challenges for the LRS operation in some regions, and even though there has been a significant drop in the cost of performing LRS, most researchers in low-income countries still find it difficult to afford [58]. Samples, therefore, continue to be sent to major sequencing centres or overseas, meaning that the results are received with considerable time delay. Portable LRS has the potential to address many of the issues that contribute to health inequity in economically disadvantaged and remote locations. Therefore, the widespread implementation of LRS with portable devices may lead to equitable global access to genomic medicine for vulnerable patients.

Another promising application of long-read sequencing and in particular nanopore sequencing has been in the critical care setting where recent reports from intensive care units (ICUs) have shown improved patient outcomes with this new technology. Long-read sequencing was shown to identify pathogens in ICU patients with serious infections with a high degree of accuracy in a timely fashion, even in cases where traditional methods fail [72]. In one report, long-read sequencing helped in the diagnosis of ventilator-associated pneumonia, which is a common complication in ventilated patients. The use of the MinION provided an accurate diagnosis within 6 h, allowing for early intervention and improved treatment outcomes [73]. LRS has also been shown to detect a broader range of pathogens than traditional methods and to provide a more detailed view of the genetic makeup of pathogens, which can be important for understanding their virulence and antibiotic resistance [74]. LRS is therefore also a promising technology for antimicrobial resistance gene detection and antimicrobial susceptibility testing and might become a vital technology for managing infections in the context of the global antibiotic resistance crisis [75].

## Challenges

While long-read sequencing technologies offer significant potential in health care, several challenges need to be addressed to enable healthcare providers to confidently adopt these technologies. One major concern is the validation of data generated through LRS. Accurate and reliable data are crucial for making informed clinical decisions. A recent study by Mahmoud et al. evaluated the accuracy of LRS platforms in detecting clinically relevant genetic variations [77]. The researchers compared LRS data with gold-standard reference methods and found that while LRS showed promise, it still had limitations in accurately detecting certain types of genetic variants. This study highlights the importance of rigorous validation processes to ensure the reliability of long-read sequencing data before its integration into standard clinical practice.

The incorporation of LRS into standard clinical practice also very much depends on the stability and reliability of the sequencing platforms. Carbo et al. evaluated the stability and performance of ONT's sequencing platform over an extended time period [78]. Their study found that while the ONT platform demonstrated high accuracy during initial testing, it showed a decline in performance over time due to technical issues and platform instability. These findings underscore the importance of ensuring the stability and consistency of LRS platforms for their successful implementation in routine clinical practice.

Furthermore, to overcome the challenges associated with LRS, it is crucial to establish standardised protocols and quality control measures. Fukasawa et al. proposed a comprehensive quality control framework for LRS data [79]. The researchers developed a set of metrics to assess data quality, including read length distribution, error rate estimation, and base quality. By applying this framework to LRS datasets, they were able to identify potential quality issues and optimise the data analysis pipeline. Establishing such standardised protocols and quality control measures will facilitate the integration of long-read sequencing into standard clinical practice by ensuring consistent and reliable data across different laboratories and platforms.

In addition to validation and stability, another critical factor for the adoption of LRS technologies in health care is their practical implementation and integration into existing clinical workflows. A recent study by Marwaha et al. evaluated the feasibility and cost-effectiveness of incorporating LRS into the diagnostic pathway for genetic disorders [80]. The researchers compared the diagnostic yield and cost per diagnosis between long-read and short-read sequencing methods. The study demonstrated that LRS had a higher diagnostic yield for complex genomic rearrangements, enabling accurate diagnosis and guiding appropriate treatment decisions. However, the study also highlighted the need for optimising workflow efficiency and reducing sequencing costs to make long-read sequencing more accessible and economically viable for routine clinical use.

Another challenge poses the data from long-read platforms which is qualitatively different from SRS data. LRS requires tailored analysis tools and bioinformatics approaches that enable to take full advantage of the technology. Consequently, various tools for base calling, error correction, de novo genome assembly, mapping, and phasing using long-read data continue to be developed [69]. A scientific community consensus on the standard algorithms and tools to process LRS data is needed but does not exist yet.

Future clinical applications, especially in regional, rural, and remote settings, will most likely require the development of highly intuitive bioinformatics analysis platforms so that clinicians are able to perform analyses without major disruption to existing clinical workflows. Regulatory, accreditation and credentialing frameworks for clinical applications close to the bedside would need review and revision, not dissimilarly to what is evolving for point-of-care ultrasound as distinct from traditional ultrasound examination and reporting.

Another limitation of LRS platforms such as nanopore is the high “cost per base” [69]. This metric is used to compare the economy of sequencing genomes across various platforms. The cost per base on the MinION has dropped recently (primarily due to a large increase in data yields) and is currently about $50 per Gb, which is similar to Illumina’s MiSeq platform, although the larger Illumina platform can achieve costs of < $10/Gb [81]. However, this metric fails to appreciate that the inherent value of a base as part of a long-read sequence is greater than the value of a base that is part of a short-read sequence. Moreover, portable ONT sequencers can be adopted without the need for a huge capital investment such as required for Illumina or PacBio sequencing.

Furthermore, with increasing research and development of LRS, it has become evident that the platform is not necessarily the limiting factor for the length of the sequenced DNA but the DNA extraction and library preparation. Production of long reads largely depends on using high‐quality DNA of high molecular weight. While saliva has been demonstrated as a reliable common non-invasive DNA source for short-read analyses, most of the library preparation protocols for LRS require high molecular weight DNA from blood or other invasive tissue collection. As the DNA once extracted from the sample is no longer bound and protected by its histones, simple disruptions and processes such as pipetting can lead to fragmenting of DNA strands to < 100 kb [76]. This ultimately reduces the potential for ultra-long-read sequencing and poses a particular challenge for remote centres where a lack of facilities and expertise may lead to DNA fragmentation during sample extraction and handling, limiting the utility of LRS. Therefore, advancements in both DNA extraction and library preparation techniques (such as the ONT VolTRAX system) will be required to reduce time as well as human error and enable widespread use of LRS [82].

**Fig. 2:**
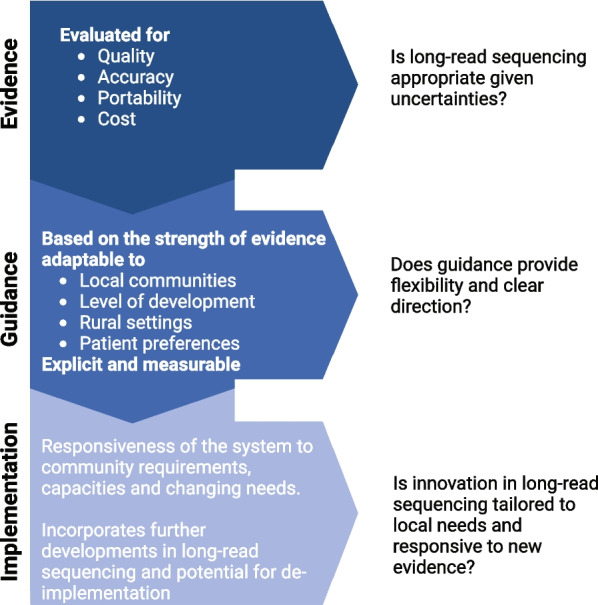
Schematic pipeline for long-read sequencing implementation in evidence-based medicine. Created with BioRender.com

Genomic testing does not only involve a laboratory testing component but also associated upstream and downstream services, including the provision of information, counselling, interpretation of test results, and clinical decision-making. Implementation of LRS-based genomic testing into a health system will therefore require consideration of these additional services in alignment with evidence-based best practices (see Fig. 2). This would include ensuring nationally consistent LRS implementation so that all patients have access to the same high-quality care. Specifically, this will require appropriate governance, an adequately prepared workforce and guidance around safety and quality of services, development of nationally consistent guidance, inter-jurisdictional and international coordination, rigorous processes for assessing the utility of LRS genomic tests, transparent decision-making, and timely monitoring and evaluation.

With the advent of LRS as a key technology for personalised medicine, healthcare services must make the decision whether to perform the testing in the community or hospital, to outsource the testing to centralised laboratories, or to create a hybrid model, where part of the testing is done on-site, and tertiary laboratories are used for data analysis and interpretation. With portable LRS devices, genomic testing has become cheaper and more user-friendly. A community-based approach would enable a much faster diagnostic turnaround and decrease the risk of losing important material or information during sample shipment. However, on-site genomic testing would require robust protocols and control of pre-analytical parameters, sample specimen selection, and sample quality.

There are ongoing collaborative national and international efforts to integrate genomic technologies into clinical and healthcare settings. Some of these efforts include Australian Genomics, Genomics England, the French Plan for Genomic Medicine 2025, the Global Alliance for Genomics and Health, and the Global Genomic Medicine Collaborative [83–85]. Currently, there are two major global priorities for implementing genomic medicine and LRS in a clinical and healthcare setting. There needs to be an evidence-based clinical application for LRS through evaluation of its long-term health and health economic impact. This will facilitate the development of standardised evaluation criteria specific to diseases and funding contexts and in relation to healthcare systems. However, common global objectives and collaborative frameworks can promote the more effective use and implementation of genomic medicine.

## Discussion and conclusions

Both nanopore long-read sequencing and SMRT sequencing have their unique advantages and applications, and choosing between these technologies depends on the specific application, associated requirements, and experimental conditions. PacBio sequencing is known for its high accuracy, making it well-suited for applications where precise SNP detection and haplotype phasing are essential. However, PacBio sequencing often requires higher DNA input amounts, making it more suitable for samples with abundant starting material.

In contrast, ONT long-read sequencing provides real-time sequencing capabilities and portability, making it advantageous in field-based or point-of-care settings. It has the ability to generate long reads with a more rapid turnaround time, enabling fast SNP detection and characterisation. Moreover, nanopore sequencing has a low input material requirement and is suitable for low-concentration analytes.

The choice between PacBio and ONT long-read sequencing depends on various factors. If high accuracy, longer read lengths, and complex genomic variations are the primary focus, PacBio sequencing is often preferred. However, if rapid turnaround time, real-time analysis, portability, and lower input material requirements are critical, nanopore sequencing can be the more suitable option.

Both, PacBio and ONT are continuously evolving, and enhancements are being made to improve their technological capabilities. Therefore, it is important to consider the requirements of a specific LRS application and monitor recent developments to determine the most suitable platform.

In addition to PacBio and ONT long-read sequencing technologies, hybrid long-read/short-read sequencing approaches have emerged as a powerful tool in genomic research. By combining the strengths of both long-read and short-read sequencing, hybrid approaches offer improved accuracy, read length, and cost-effectiveness.

Long reads provide information about complex genomic variations, while the short reads offer high accuracy for base-level resolution and enable efficient error correction. This combination of technologies is particularly valuable for de novo genome assembly, where the long reads help in resolving repetitive regions and complex structural variations, while the short reads aid in accurate consensus generation. Additionally, hybrid sequencing can be beneficial in studying complex genomes, such as those with high levels of heterozygosity or polyploidy, where accurate phasing and haplotyping are essential.

Hybrid long-read/short-read sequencing approaches continue to evolve, with improvements in protocols, library preparation methods, and bioinformatics tools. These advancements enhance the quality and utility of the generated data, making hybrid sequencing an increasingly attractive option for a wide range of genomic applications.

Overall, the choice between short-read, long-read, or hybrid sequencing depends on the specific research goals, experimental conditions, and technological advancements. Staying informed about the latest developments in sequencing technologies and understanding the unique advantages of each approach is crucial for making informed decisions in genomic research.

LRS technologies such as nanopore and SMRT sequencing are opening new exciting avenues in the diagnosis and treatment of genetic disorders, cancer and infectious diseases. To integrate this technology in health care, funding is required for sequencing devices, technology development, workforce upskilling, and increased integration between academic centres of research excellence, hospitals and health services. The associated rapid scientific and technological developments will require an environment where healthcare providers work closely together with highly specialised physicians and clinical molecular geneticists who are supported by experts in laboratory medicine, genomics, and bioinformatics. Once the current limitations such as costs, complexity and amount of data, availability and lack of clinical validation are overcome, it is widely expected that LRS will become the breakthrough technology and standard of care for genomic analysis, allowing a widespread implementation of precision medicine in a large number of clinical and remote settings.

## Abbreviations

AMR: Antimicrobial-resistant
CCS: Circular consensus sequencing
GVHD: Graft-versus-host disease
ICU: Intensive care unit
KIR: Killer cell immunoglobulin-like receptor
HLA: Human leukocyte antigen
LRS: Long-read sequencing
MPXV: Monkeypox virus
ONT: Oxford Nanopore Technology
SMRT: Single-molecule real-time
SRS: Short-read sequencing
STI: Sexually transmitted infection
STR: Short tandem repeats
SV: Structural variants
WGS: Whole-genome sequencing
